# Methodology and recruitment for a randomised controlled trial to evaluate the safety of *wahakura* for infant bedsharing

**DOI:** 10.1186/1471-2431-14-240

**Published:** 2014-09-28

**Authors:** David Tipene-Leach, Sally Baddock, Sheila Williams, Raymond Jones, Angeline Tangiora, Sally Abel, Barry Taylor

**Affiliations:** Women’s and Children’s Health, Dunedin School of Medicine, University of Otago, PO Box 913, Dunedin, New Zealand; School of Midwifery, Otago Polytechnic, Dunedin, New Zealand; Preventive & Social Medicine, Dunedin School of Medicine, University of Otago, Dunedin, New Zealand; Kaupapa Consulting Ltd, 52 Vigor Brown St, Napier, New Zealand

**Keywords:** Sudden Unexpected Death in Infancy, Sudden Infant Death Syndrome, Infant, Sleep, Prevention, Culture, Protocol, Indigenous, Bedsharing, Co-sleeping

## Abstract

**Background:**

Sudden Unexpected Death in Infancy (SUDI) has persistent high rates in deprived indigenous communities and much of this mortality is attributable to unsafe sleep environments. Whilst health promotion worldwide has concentrated on avoidance of bedsharing, the indigenous Māori community in New Zealand has reproduced a traditional flax bassinet (*wahakura*) designed to be used in ways that include bedsharing. To date there has been no assessment of the safety of this traditional sleeping device.

**Methods/Design:**

This two arm randomised controlled trial is being conducted with 200 mother-baby dyads recruited from Māori communities in areas of high deprivation in the Hawkes Bay, New Zealand. They are randomised to *wahakura* or bassinet use and investigation includes questionnaires at baseline (pregnancy), when baby is 1, 3, and 6 months, and an overnight video sleep study at 1 month with monitoring of baby temperature and oxygen saturation, and measurement of baby urinary cotinine and maternal salivary oxytocin. Outcome measures are amount of time head covered, amount of time in thermal comfort zone, number of hypoxic events, amount of time in the assigned sleep device, amount of time breastfeeding, number of parental (non-feed related) touching infant events, amount of time in the prone sleep position, the number of behavioural arousals and the amount of time infant is awake overnight. Survey data will compare breastfeeding patterns at 1, 3, and 6 months as well as data on maternal mind-mindedness, maternal wellbeing, attachment to baby, and maternal sleep patterns.

**Discussion:**

Indigenous communities require creative SUDI interventions that fit within their prevailing world view. This trial, and its assessment of the safety of a *wahakura* relative to a standard bassinet, is an important contribution to the range of SUDI prevention research being undertaken worldwide.

**Trials registration:**

Australian New Zealand Clinical Trials Registry: ACTRN12610000993099 Registered 16^th^ November 2010

## Background

Sudden Unexpected Death in Infancy (SUDI) is the biggest single component of post neonatal death in the developed world. The unexplained portion of these deaths, typically called Sudden Infant Death Syndrome (SIDS), has been defined by the sudden death of an infant in sleep, which is unexplained after the review of the clinical history, post-mortem findings, and examination of the circumstances of death [1]. The term SUDI was developed to include causes of death such as “positional asphyxia” and “undetermined”, which are often used when known risk factors are present even though the contribution of the risk factors to death is unclear. The term SUDI encompasses SIDS and these more uncertain scenarios [2] and reflects the increasing focus on identifying and reducing unsafe sleep environments as a strategy to reduce post-neonatal mortality [3].

### SUDI

In New Zealand, the SUDI mortality rate over the 2003–2007 period was 1.1 per 1000 live births, with between 50–85 babies dying annually [3]. Sixty two percent of these deaths occurred in the indigenous Māori community, who comprised only 15% of the population. The SUDI rate for Māori during this time was 2.34 deaths per 1000 live births; that is, five times the rate of European New Zealanders (non-Māori, non-Pacific, non-Asian) at 0.52 deaths per 1000 births [3]. Indigenous peoples in other countries have similar disparities, for instance, Native American and Alaskan populations in the United States [4], Inuits in Nunavut, Canada [5], and Western Australian Aboriginals [6] have SUDI rates between 3–8 times the rates of their non-indigenous counterparts.

SIDS rates decreased markedly around the world with the introduction of the back sleeping position; in New Zealand, SIDS rates fell from 4.4 per 1000 live births in 1988 [7] to 1.6 per 1000 live births in 2002 [3]. The peak age for SUDI in New Zealand moved from three months of age in the 1990s to one to two months of age in the period, 2003–2007. Similar trends were identified elsewhere [8]. Over this time there was also a marked widening of disparities between social groups and SUDI became increasingly associated with poverty, poor education and maternal smoking [8–10].

### Bedsharing

Many case control studies have identified conditions in which bedsharing is associated with increased risk of sudden infant death. The most consistent finding is that exposure to cigarette smoke in utero greatly increases the risk of SIDS when bedsharing [11, 12]. Other contributing factors include excessive maternal tiredness, infant overheating and household overcrowding [12], the use of sedative drugs [13], or alcohol [14, 15] and maternal obesity [16]. In the absence of any of these other factors, there is a suggestion that although bedsharing per se has some risk for babies under the age of 14 weeks [15], the risk is significantly smaller than where there is smoking during pregnancy.

### Smoking

Maternal smoking in pregnancy is recognised as the primary cause of increased vulnerability when bedsharing. A decreased arousability to hypoxia from fetal exposure to passive smoking may well be the mechanism that leads to this increased risk [17–19]. To add to the complexity, bedsharing is also a common and valued childcare practice in many cultures, including Māori and Pacific families in New Zealand [20] and is seen in many cultures as developing and maintaining a sense of ongoing connection to the infant [21, 22] as well as facilitating breast feeding [23–25]. Māori women however, have high rates of smoking and Māori women from communities of high deprivation in Auckland, New Zealand’s biggest city, have a prevalence of cigarette smoking in pregnancy of 53% [26]. Despite vigorous efforts to decrease smoking in pregnancy [27] there has been limited success [28]. Likewise, efforts by health professionals, the Ministry of Health and coroners to discourage bedsharing do not appear to have had any impact as 65% of Māori mothers in Auckland bedshare with their infants for some part of the night [26]. In addition, a mortality review of the years 2000 to 2009 in the same city showed that 64% of all SUDI cases were found dead in a shared bed [29]. Nationally, 43% of SUDI deaths occurred when bedsharing [3], and an increasing proportion of these deaths are now labelled accidental suffocation [30]. Unfortunately, mortality review data collection around risk factors associated with these deaths is incomplete in New Zealand and it was not possible to identify the contribution of factors such as smoking in pregnancy or alcohol consumption on the night of death.

### A culturally derived intervention

In response to this combination of high risk behaviours and the cultural value of bedsharing as an important component of infant care practices [31–33], the Māori community has developed the *wahakura*. The *wahakura* is a woven flax bassinet with a thin, firm mattress designed specifically to create a separate sleeping surface in the shared sleeping space. It is distributed with a set of ‘safe sleeping rules’ [34] derived from the recommendations of the New Zealand Ministry of Health. Its acceptability to Māori comes from its community origin and its Māori nature and appearance [35].

*Wahakura* are increasingly being used by families across the country and in some places are distributed by District Health Boards [36], and thus there is an imperative to establish their safety profile. To date, there are no studies of the effect of the use of the *wahakura* in the context of bedsharing on infant or adult sleeping behaviour; nor of what effect the professional interactions associated with the handover of *wahakura* might have on wider parental behaviour like the recall of safe sleeping advice, parental response to infant needs, changes in smoking behaviour or attachment behaviours; or of the *wahakura* on infant behaviour (e.g. breastfeeding, sleeping pattern).

Furthermore, as we learn more about the complex interaction between biology, environment and culture, and how mothering is often at the intersection of these concepts, a fuller understanding of the effects of such interventions would usefully measure some of these variables including salivary oxytocin levels [37, 38], ‘mind-mindedness’ (the mother’s ability to think about her infant’s emotions, thoughts and needs) and post-natal mood, all of which have important effects on parenting behaviour [38–40].

### Aim of the study

To determine the safety and other benefits, or harm, from providing either a *wahakura* or bassinet to families attending a mainly Māori midwifery service from geographical areas of high deprivation in an urban setting in New Zealand. We intend to compare physiological and behavioural measures of infants in the two sleep environments, such as temperature and desaturation events, differences in infant head covering events and breastfeeding time and whether allocation of sleep device impacts on time spent bedsharing.

In addition, we will look at how the use of a *wahakura* relates to other issues of safety in the infant period, to ‘mothering’, mind-mindedness, a sense of family, maternal post-partum depression and to cultural identity.

## Methods/Design

### Overall study design

Ethical approval to conduct this study has been granted by the New Zealand Central Region Ethics Committee (CEN/10/12/054).

This is a randomised controlled trial of *wahakura* versus bassinet to test the following hypotheses:

1. That the use of a *wahakura* is not significantly different in terms of thermal environment, head covered duration, episodes of oxygen desaturation, or total sleep duration, than infants sleeping in a separate bassinet in the same room.
2. That the ‘*wahakura* group’ spends less time per night bedsharing on the same bed surface than the ‘bassinet group’.
3. That the use of a *wahakura* is associated with significantly increased breastfeeding episodes, breastfeeding duration, and more parental “looking and touching” episodes.
4. That use of a *wahakura* is associated with greater involvement of the extended family, more attention to other issues of safety, and a greater sense of connection to family
5. That the use of the *wahakura* promotes maternal and extended family mind-mindedness and development of individual, family, and cultural identity.

Participants will be randomized to receive a *wahakura* or bassinet and the above aspects will be studied using a combination of questionnaires and an overnight sleep study with video, temperature and oxygen saturation measurement. Baby urine (for cotinine) and maternal saliva (for oxytocin) will be collected, and a recording will be made of mothers talking about their feelings about their baby.

### Participants and recruitment

Recruitment and data collection will be done in Hawke’s Bay, a region in the North Island of New Zealand with two urban areas, Hastings and Napier. Mothers booking into two mainly Māori midwifery services will be informed of the study by their midwife, and asked if they wish to participate.

Should the mother express interest, then the study researcher will either meet her at the clinic venue or visit her at home to explain the study in more depth, offering further time to discuss the study with the extended family. An information sheet directly aimed at the extended family, written in an appropriate English and Māori format will describe the study. Should the mother agree to be involved, written informed consent will be obtained and baseline questionnaires completed. Participants will be randomised to a sleeping device (randomisation in blocks by parity and deprivation quintile), with either a *wahakura* or a bassinet given to the family. Anonymous demographic information (age, ethnicity, parity and deprivation score) [41] will be collected for those who decline to participate in the study.

**Figure 1:**
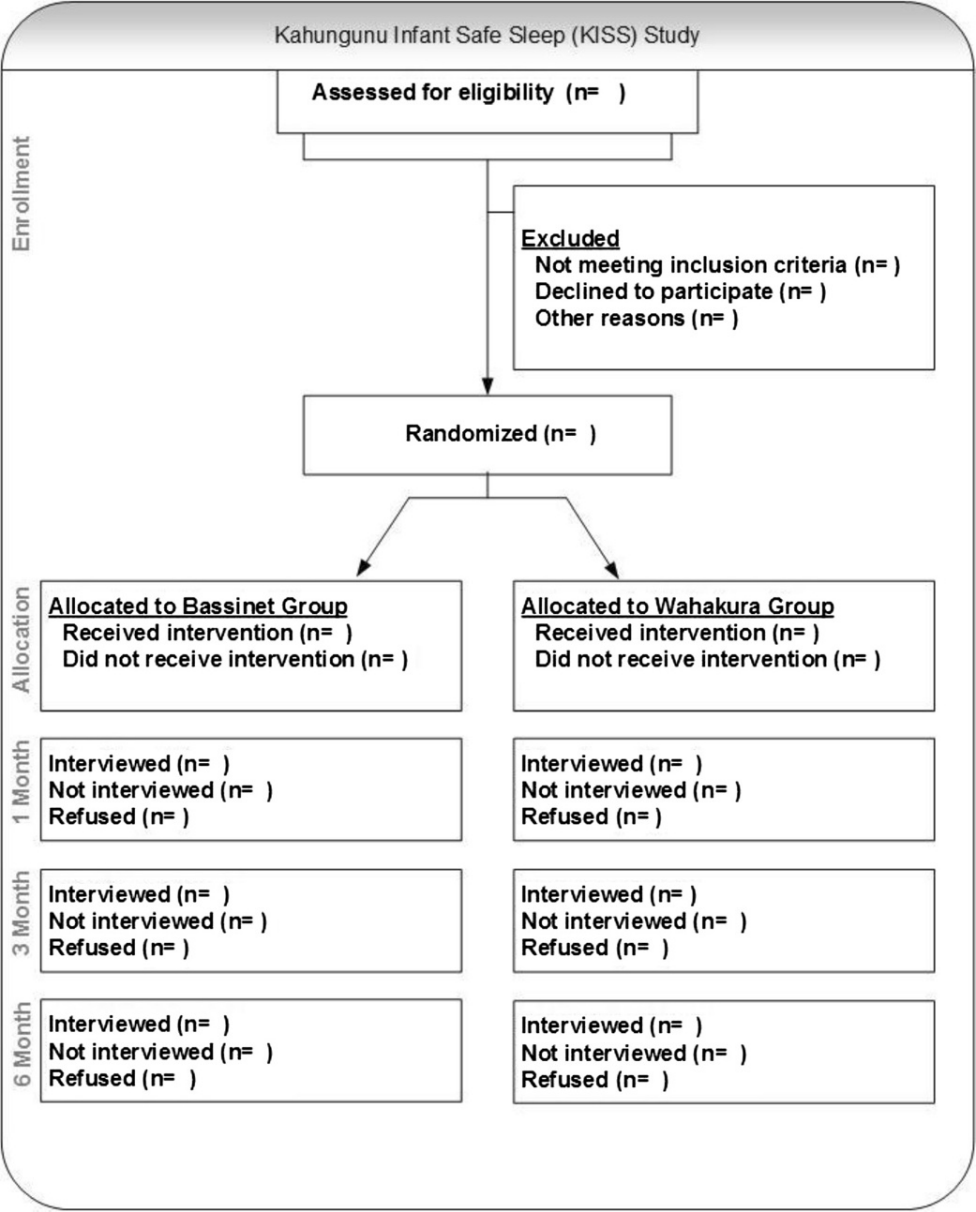
**Consort diagram for Kahungunu infant sleep study.**

A birth congratulations card will be sent to the family shortly after the birth of the baby as a reminder to use the appropriate sleep device, and telling them that they will be contacted to organise a home sleep study when the baby is 1 month old. Participants will be given a $50 grocery voucher gift after the 1 month sleep study, and a $25 voucher on completion of each of the 3 month face to face interview and the 6 month telephone interview (see Figure 1).

### Eligibility

Eligible participants are all women booking for antenatal care from two midwifery practices working with mainly Māori women from low socio-economic areas in the Hawke’s Bay, who are resident in the Hawkes Bay District Health Board, and likely to remain in that area for at least six months.

### Exclusion criteria

Babies born <36 weeks gestation, <2500 g birth weight, those admitted to the neonatal intensive care unit (NICU) for >3 days and those with severe congenital anomaly will be excluded. Mothers with a previous unexplained sudden infant death, who have severe mental health problems (as determined by contact with mental health services) or who are involved in a methadone maintenance programme will also be excluded.

### Randomisation

Mothers agreeing to participate will be randomised to one of the two groups. Allocation will be concealed and performed, following application of inclusion/exclusion criteria and consent to participate in the study, by opening a sealed envelope opened in numbered sequence. As level of deprivation and parity may significantly affect the primary outcomes, stratified block allocations will be used employing a block size of 3 within each strata combination. Deprivation quintile (derived from home address) of less than 3 versus 3 or more, and parity of 1 versus 2 or more will be used.

After allocation, participant blinding will not be possible. However, the analysis of all overnight studies will, where possible, be performed blind to group allocation. Any measures of parenting that are recorded by audio, will then be analysed blind to group allocation.

### Contamination

There may be contamination with those assigned to one condition deciding to obtain and use the other type of sleeping device. We will check the degree of contamination by identifying this at the 1 month sleep study, and by asking at each time point about use of other sleep devices. For safety analyses (head covering, peripheral temperature and oxygen saturation), analysis will be by actual device used. For breast-feeding results, both the intention to treat and actual sleep device used will be analysed.

### Sleep devices

#### Wahakura

The *wahakura* is a 36 × 72 cm flax bassinet, with no handles, and a 20 mm thick foam sponge mattress covered in a cotton pillowcase. They will be woven by a local weavers’ group, and supplied to the mother during the pregnancy, with some standard instructions as below.

#### Bassinet

A portable standing bassinet, custom designed in New Zealand for distribution to infants at high risk, will be used. This bassinet can easily be moved and transported in a car. The base will contain an identical 20 mm foam sponge mattress as used in the *wahakura*.

#### Safe sleep instructions

We recommend that babies always use the assigned sleeping device as their sleep place and, noting the portability of both devices, that the sleeping device is passed on to all carers (babysitter, grandparents, and extended family). *Wahakura* are to be used for every location where the baby sleeps. That is, it may be placed on the floor, on a mattress, on a flat couch, in a shared bed, or in a cot.

Babies should sleep on their back, with no pillows, face always clear of blankets, with no toys or loose objects in the sleeping environments. We recommend that direct bedsharing, that is bedsharing without a *wahakura or other protective device,* should be for cuddles and feeding only, and that the baby be put back into the *wahakura* or bassinet for sleeping.

Some options for safe bedding will be suggested for both the *wahakura* and the bassinet, for example, the sleeping sack (baby sleeping bag), or, as is usual for a bassinet, the blanket threaded under the mattress and wrapped over baby and then tucked in. Mothers will be routinely supplied with a Safe Sleep brochure outlining the above Safe Sleep messages.

### Outcome measures

Primary outcome measures for the infant will be derived from the overnight sleep study (amount of time head covered, amount of time in thermal comfort zone, number of hypoxic events and amount of time in the sleep device) and from surveys (full or exclusive breastfeeding at 3 and 6 months). Secondary outcomes identified from the sleep study include number of infant head covering events, number of parental (non-feed related) touching infant events, amount of time in the prone sleep position, the number of behavioural arousals, and the amount of time infant is awake overnight.

Psychosocial factors will also be measured. Using questionnaires, maternal environmental chaos will be measured at baseline. Social support, and social and economic stress will be measured at baseline and 3 months. Maternal depression and change in depression (from baseline) will be measured at 3 months and 6 months. Parenting factors, including beliefs about infant care, attachment, and parenting adaptation will be measured at baseline and 3 months using both the questionnaires and maternal salivary oxytocin levels at baseline, 1 month and 3 months. Maternal mind-mindedness will be measured at baseline, 3 and 6 months, using the questionnaires, and an audio recording of the mother talking about her baby (see Table 1).

**Table 1:** **Outline of measures and when they will be performed**

	Age of child			
	Pregnancy	1 month	3 months	6 months
Maternal and family demographics^1^	X	-	-	-
Maternal smoking status	X	X	-	-
Maternal alcohol and recreational drug use^2^	X	X	-	-
Baby urinary cotinine		X		
Maternal salivary oxytocin and cotinine	X	X	X	-
Perinatal variables	-	X	-	-
Sleep study	-	X	-	-
Breastfeeding status	-	X	X	X
Dummy use	-	X	X	X
Frequency and duration bedsharing	-	X	X	X
Use of well-child/primary care and secondary care services	-	X	X	X
Environmental chaos^3^	X	-	-	-
Social support^4^	X	-	X	
Social and economic stress^5^	X	-	X	X
Maternal depression^6^	X	-	X	X
Beliefs about infant care^7^	X	-	X	-
Attachment and parenting adaptation^8^	X	-	X	-
Mind-mindedness^9^	X	-	X	X

^1^Family structure, maternal education, combined family income, ethnicity, type of accommodation, number of bedrooms.

^2^Derived from the Australian national drug strategy household survey [42] and Australian health survey 2001 [43].

^3^Confusion, Hubub and Order Scale [44].

^4^NSW Child Health Survey [45].

^5^Measures used in POI.nz study (original source lost) [46]. Economic stress as utilised by the Welsh Family & School Transition Project 2001 (Harold, G., Personal Communication, June, 2007).

^6^Edinburgh Postnatal Depression Scale [47].

^7^Questions designed by the authors to measure beliefs about infant care.

^8^Attachment and Adaptation Scales from the Parenting Stress Index [48].

^9^Questions designed by the authors to measure mind-mindedness. We will also code a 5 minute audio recording of each mother talking freely about her baby.

### Sample size

Our previous studies comparing bed-sharing to cot-sleeping infants [49, 50] have been used to determine sample size using two of our primary outcome measures. The third major outcome (breast-feeding proportion) is derived from national data on breast-feeding (full or exclusive) by ethnicity [51].

### Head covering

The proportion of babies having an overnight sleep with a head covering episode by blankets was 1/37 and so, in order to detect a difference of 15% between babies sleeping in wahakura and cots with 80% power using the 5% level of significance, two groups of 88 babies would be needed.

Assuming the proportion of babies having an overnight sleep with a head-covering episode by blankets will be 3% when sleeping in a bassinette, 88 babies per group are required to detect a difference of 15% between babies sleeping in wahakura and cots with 80% power using the 5% level of significance.

### Temperature control

From our previous study [49], we estimated that 32% of bedsharing babies and 3.5% of cot sleeping babies had a core-peripheral temperature difference of <1°C after they had been asleep for 3.5 hours, suggesting thermal stress from a warm environment. About 40 babies per group are needed detect a difference of this magnitude with 90% power using the 5% level of significance. Ideally, babies should be neither too hot nor too cold; 75% of the cot-sleeping babies were in the ideal comfort zone, where the peripheral temperature is between -1°C and -3°C of the core temperature. Two groups of 98 babies have the potential to detect a difference of 20% with 80% power using the 5% level of significance.

### Breastfeeding

Currently, 45% of Māori babies are still breastfeeding at 3 months. In order to show a 20% increase in breastfeeding at 3 months (to 65%), 106 babies would be needed in each group to have 80% power using the 5% level of significance.

### Drop-out rate

After discussion with the midwifery group within which we will work, we estimate dropout of 5% at 1 month, 20% at 3 months, and 25% at 6 months. Our power studies are mainly dependent on the 1 month measures, so we will increase our required numbers by 5%.

### Total numbers required

Overall, we believe that we should aim to recruit enough babies so that we have complete data at 1 month, of 100 babies per group – i.e. enrol 105 babies per group.

### Data collection and transfer

A unique feature of this study is that it assesses a Māori-derived intervention and that recruitment and data collection will occur in an area of New Zealand with a high Māori population, with high levels of deprivation, and by researchers who are Māori with connections to the local community.

Data files of 20 Gbytes/study will be transfered from the study area to the University of Otago using the New Zealand eScience infrastructure (NeSI) high speed KAREN network. The Autonomy TeleForm system (Autonomy Inc, San Francisco, CA94105, USA) will be used to create machine-readable survey forms. Completed surveys will be scanned and sent electronically to the system which will store the information in a customised database. This automated data entry is designed to reduce human error.

### Measures

#### Sleep studies

A formal home sleep study will be completed when babies are 1 month old. After negotiation with the family, a researcher will visit the home in the evening, and set up the equipment for recording. We plan to use the minimum possible equipment, with the least possible attachments to the baby. Infrared video using a Swann wireless ADW-400 digital camera and recorder, and oximetry using a Massimo Rad 8 set to a 2 second averaging time (analysed using Visi-Download software) will be recorded. Four temperature probes will be used to measure infant toe and core temperature (measured over the liver) and room and outside temperature, using a flat film RTD temperature sensor (5×2 mm). Temperature will be recorded onto GP-HR general purpose 4 channel logger to be attached to the Massimo oximeter.

Video recordings will be viewed off-line using Noldus Observer XT, a software package for the collection, analysis, and presentation of observational data. The data will be coded according to a taxonomy adapted from that used in the Durham University Parent-Infant Sleep Lab (personal communication, Prof. Helen Ball, Parent-Infant Sleep Lab, Durham University; June 2012). Key categories will include infant sleep time, infant awake time, behavioural arousals, head covering events, breastfeeding events, infant sleep position, and maternal interactions. The software allows synchronisation with physiological recordings of oxygen saturation and temperature. Hypoxic events will be defined as decreases to <90% lasting >10 seconds. Infant peripheral temperature will be used as an indicator of thermal comfort. On the basis of our previous studies [49] we decided to use a peripheral temperature of <34 degrees as indicating cold stress, or >36 degrees as indicating heat stress. The data will be analysed to identify behavioural events associated with hypoxic episodes or changes in infant temperature.

#### Breastfeeding

Breastfeeding is a primary outcome for this study and information will be gathered from the questionnaires and the overnight sleep study. Participants will be asked at the baseline survey about their knowledge of breastfeeding and their intention to breastfeed. At 1, 3 and 6 months, participants will be asked questions about breastfeeding from which it will be possible to describe the frequency of breasfeeding, full, exclusive or partial breastfeeding, when breastfeeding ceased, and/or when solids or other milk were introduced. They will also be asked about their mothers’ and their partners’ support for, and attitudes toward, breastfeeding. The duration and timing of the breastfeeding episodes will be identified from the sleep study, as well as head covering events during breastfeeding.

#### Biological samples

To measure passive infant exposure to cigarette smoke (cotinine levels), urine will be collected at 1 month during the sleep study by placing a cotton wool ball into the nappy of the baby at each nappy change. Between 3–6 such cotton wool balls will be collected and placed in a 20 ml syringe which will then be squeezed forcing a urine sample into an appropriate container.

To measure salivary oxytocin, [37, 38], saliva samples will be collected at the baseline, 1 and 3 month visits after 24 hours of abstention from alcohol and salty foods, no brushing or flossing of teeth for 24 hours, and having not consumed drinking water immediately before collecting the sample. Saliva will be gathered in the mouth and then extruded into the appropriate laboratory container via a standard drinking straw.

The saliva samples will be spun, recollected into two cryotubes, frozen on the same day and stored in a local laboratory until delivered to the testing laboratory for analyses of oxytocin. The sample in the second cryotube will be stored until analysed for cotinine. The urine samples will be directly frozen at -40 degrees Celsius until they are analysed for cotinine.

#### Psychological measures

In addition to biological factors, a number of psychosocial factors can influence parenting and infant psychological wellbeing. For example, maternal depression [40], stress [52, 53] and environmental chaos [44] negatively impact infant outcomes, whereas a mother’s mind-mindedness, and the quality of the maternal-infant attachment, predict positive infant outcomes [54–56]. Data gathered during the questionnaires administered at pregnancy, 3 months, and 6 months, will be used to measure some of the effects of this culturally appropriate intervention on maternal and infant psychological outcomes.

To measure environmental chaos, the Confusion, Hubbub and Order Scale will be administered at baseline [44]. At baseline and 3 months, social and economic stress will be measured using the questions from the Welsh Family & School Transition Project 2001 (Harold, G., Personal Communication, June, 2007), and social support will be measured using questions from the NSW Child Health Survey [45]. Maternal depression will be measured using the Edinburgh Postnatal Depression Scale [47], which will be administered at baseline, 3, and 6 months. Parenting factors will be measured at baseline and 3 months, using questions designed by the authors to measure beliefs about infant care (e.g., 1 = Babies benefit from someone staying with them as they settle to sleep, 5 = Babies benefit by learning to sleep by themselves), as well as items from the Attachment and Adaptation subscales of the Parenting Stress Index [48]. Maternal mind-mindedness will be measured at baseline, 3, and 6 months, using Likert scale questions designed by the authors (e.g. Baby has his/her own thoughts, 1 = all the time, 5 = None of the time), derived from mind-mindedness research [57–60]. Maternal mind-mindedness and attachment will also be assessed during pregnancy and at 3 months, by coding a 5 minute audio recording of the mother talking freely about her baby in response to the question, “Could you please tell me what thoughts you have has so far about your (unborn) baby?” The coding system will be based on previous mind-mindedness and attachment research [57–60].

#### Data analysis

The data will be analysed using modified intention to treat, including only those who completed at least one of the 1, 3 or 6 month visits. Chi-squared tests or Student t-tests will be used to compare the outcomes in the two groups. A per protocol analysis will also be carried out to estimate differences in the outcome variables in those who were compliant with the sleeping arrangement to which they were assigned.

This study will also provide an opportunity to examine factors associated with breastfeeding and sleep practices in a disadvantaged group. The breastfeeding data will be analysed using a discrete time model.

## Discussion

As far as we are aware, there are no other studies of this nature being performed anywhere in the world. SUDI prevention is an important area of research in the Western world, and the advent of ongoing, extremely high rates of SUDI in communities of deprivation, including indigenous communities, has not been effectively addressed to date. In addition, the *wahakura* has not been developed by the scientific community, nor has it arisen from the wider health sector. It is a cultural reclamation of an item used traditionally in Māori society in the effort to continue the valued practice of bedsharing, while maintaining the safety of the infant. We will assess the risks and benefits around the *wahakura*, to establish whether it might be able to be recommended as a safe sleeping device for prevention of SUDI. Whilst it has been suggested there is some ‘face validity’ [61] and there is support in the high risk community for this device, there is no empirical evidence as yet assessing its safety. This study seeks to provide exactly that.
